# Simultaneously performed off-pump coronary artery bypass grafting and colectomy: a case report

**DOI:** 10.1186/1477-7819-8-50

**Published:** 2010-06-15

**Authors:** Panagiotis Dedeilias, Ioannis Nenekidis, Efstratios Koletsis, Nikolaos G Baikoussis, Panagiotis Hountis, Dimitrios Exarhos, Serafim Klimopoulos

**Affiliations:** 1 1st Cardiac Surgery Department, "Evangelismos" General Hospital, Athens, Greece; 2 Department of Cardiothoracic Surgery, University of Patras School of Medicine, Patras, Greece; 3 2nd Surgery Department, "Evangelismos" General Hospital, Athens, Greece; 4 Department of Radiology, "Evangelismos" General Hospital, Athens, Greece

## Abstract

This is written so as to report the case of a 71-year-old male with a diagnosis of sigmoid adenocarcinoma accompanied by severe coronary artery disease and unstable angina, which was subject to simultaneous surgical treatment. The patient initially underwent an off-pump coronary artery revascularization in order to avoid the complications of cardiopulmonary bypass, providing the opportunity of a colectomy at the same time with the use of safe surgical means. Our case suggests that performing an off-pump bypass procedure prior to cancer surgery can be an appropriate course of action in carefully selected cases.

## Introduction

Up to now, patients being surgically treated at the same time for two non related reasons was not a common practice in medicine. On the other hand the morbidity records, of a combined surgical approach such as a heart operation prior to digestive surgery, are acceptable, though anticipating the ability of the patient to overcome the complications of a double surgical procedure. However, there is some skepticism regarding the effect of the extracorporeal circulation (ECC) on the expansion of the coexisting malignancy. When both pathologies are acute, sometimes there is not adequate time to spend on deciding which disease calls for more immediate action. In general, we place priority upon coronary revascularization to oncologic procedure for it is more life-threatening. We are describing the simultaneous surgical procedure of a patient with hemorrhagic left colon cancer combined with severe coronary artery disease.

## Case Report

**Figure 1:**
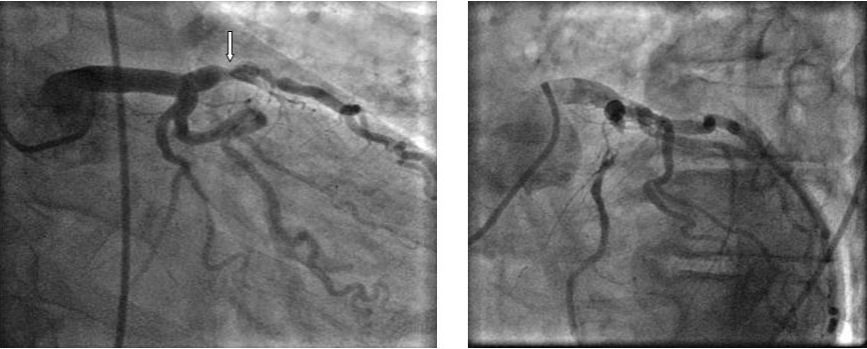
**The preoperative patient's coronary angiography showing the significant stenosis of the (LAD), (arrow)**.

**Figure 2:**
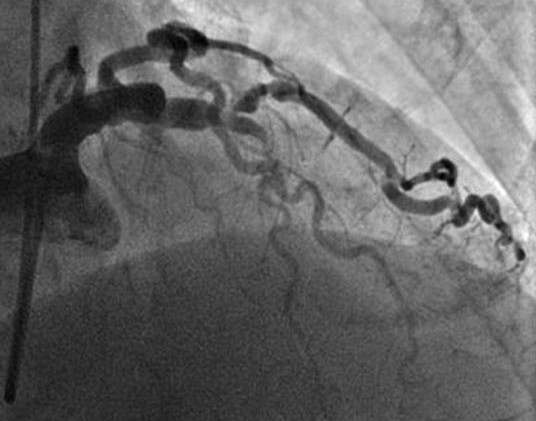
**In this figure is revealed the stenosis of the first diagonal (DG1)**.

**Figure 3:**
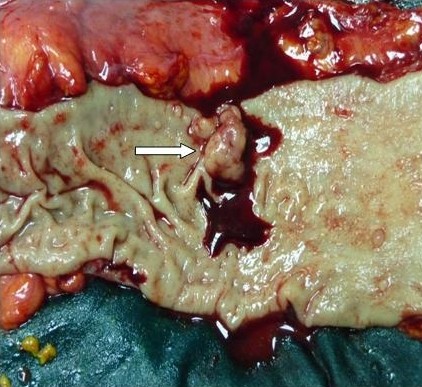
**Macroscopic appearance of the resected colon cancer (arrow)**.

A 71-year-old man was admitted to the intensive care unit of our hospital with chest pain, progressive dyspnoea and bleeding from the left colon. His medical history pointed out that a month ago he suffered from an acute myocardial infarction treated conservatively without any intervention. Following this ischemic episode another incidence of bleeding from the lower intestinal tract took place, a fact that created a more complicated clinical condition. Further investigation and biopsy revealed the presence of cancer at the recto-sigmoidal portion of the large intestine. The rest of the anamnesis did not show any other pathology or previous operations. At the time of admission the electrocardiogram showed anterior and lateral myocardial ischemia accompanied by an elevation of myocardial enzymes (CK = 570 IU/L, CK-MB = 101 IU/L, Troponin = 0,54 ng/mL and Pro-BNP = 1458 Units). The rest of his biochemical profile was normal but the hematocrit was low (Hct: 30.7%) probably due to current blood loss by colorectal malignancy. Other elements of blood count and coagulation time were within physiologic values. After a day of intensive treatment, a coronary angiography was performed by the cardiologists. A total occlusion of the left anterior descending artery (LAD) just before the origin of the first diagonal branch was revealed. In addition, a critical stenosis (95%) of the first large diagonal (DG1) was found (Figure 1,2). Angioplasty attempted at the time was interrupted due to hemodynamic instability of the patient and electrocardiographic changes. Added to that, the percutaneous angioplasty could not be adequately protected with prompt antiplatelet therapy due to colorectal bleeding. The patient then was referred to cardiac surgeon for surgical consideration. A preoperative staging of colon cancer was performed (Total body CT scanning, radionuclide bone scanning, gastroscopy, tumors markers), revealing no sites of metastasis. After taking into consideration, the necessary preoperative information, the surgical board, consisting of cardiovascular and general surgeons, decided to proceed to a simultaneous handling of the aforementioned pathologies. After the induction of anaesthesia a double off pump coronary artery bypass was performed within 125 minutes. Under fool systemic heparinization the left internal thoracic artery (LIMA) was anastomosed to the left anterior descending artery (LAD) and a saphenous vein graft (SVG) was placed on the first diagonal (DG1). During surgery a meticulous hemodynamic monitoring through Swan-Ganz catheter and INVOS cerebral oximetry was done. Sternotomy was closed and dressed very accurately in order to avoid infective complications like mediastinitis. Consecutively we subtracted via a subumbilical incision the diseased colon by a lower anterior recto-sigmoidal resection. The tumour included within the resected intestine was 2,5 × 1,5 × 2 cm in size. The specimen was resected within healthy limits and macroscopically there were no perceptible infiltrations. The quick biopsy (extemporaneous) was positive for malignancy (figure 3). Intraoperatively, the patient was kept in normothermia and received two units of red blood cells. After a total surgical time of 235 minutes he was admitted to the intensive care unit without inotropic support. Tracheal extubation was achieved 10 hours later. He was transferred to a ward the following day and was discharged, on the 10^th ^postoperative day after an uneventful course. Histopathological findings were indicative of a well-differentiated intramucosal intestinal adenocarcinoma with multiple non infiltrated lymph nodes (Classification according to WHO: T1N0M0, Stage A according to Duke).

## Discussion

**Figure 4:**
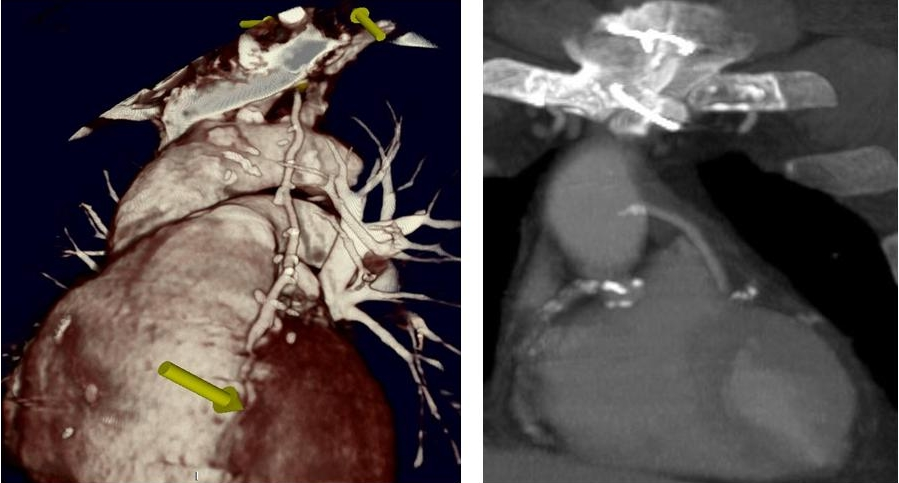
**Postoperative Reconstruction CT Coronary Angiography (LIMA on LAD and SVG on first Diagonal)**.

As a supreme tool the off pump technique of coronary artery bypass grafting (OPCAB), facilitated the one stage cardiac revascularization and colectomy to be done safely at the same time. In the beginning we avoided the effect of extracorporeal circulation to the progression of the coexisting malignancy by using the off-pump coronary bypass technique. However, it is suggested that the heart-lung machine (HLM) as well as the cell saver devices (CS) can be used safely with patients with malignant disease and do not increase any risk of hematogenous dissemination in cases of malignancy coexistence [1]. It is of great importance to mention that late complications of extracorporeal circulation contribute indirectly to the expansion of the existing malignancy according to the international bibliography [1-3]. What really matters is that the inflammatory response of the cardiopulmonary bypass (CPB) and the immunosuppression phenomenon, as consequences, play a significant role in the augmentation of the oncologic disease [3,4]. The systemic inflammatory response provoked by cardiopulmonary bypass is briefly combined with, postoperative fever, increase of capillary permeability, accumulation of interstitial fluid as well as leukocytosis [3]. The innate and adaptive features of the immune system are severely affected by the HLM [4]. CPB activates the complement system due to the bioincompatible nature of the surfaces encountered in the CPB apparatus and the release of endotoxin into the systemic circulation [5,6]. Neutrophils are activated [7] and also total lymphocytes, T-lymphocytes, B-lymphocytes, natural killer cells and helper T-cells show significant decrease in absolute numbers for more than one week postoperatively as compared to the preoperative values [8]. The weakness of the immune system caused by the CPB might affect the spread of the coexisting tumor [2,9]. Multiple metastases of lung cancer have been reported in patients with lung cancer being subject to cardiac surgery in the conventional way and with the use of HLM [9,10]. The same hypothesis is adopted by other authors, indicating that the combined cardiac and oncologic procedures should be performed avoiding the CPB [2,10-13]. Thus a mixture of immune activities caused by the CPB might affect the spread of the coexisting malignancy. The patient postoperatively remained uneventful and during the first 8 months of postoperative convalescence, showed no signs of recurrence. According to our knowledge such a case belongs to a limited group of combined operations ever published in literature. These results support the effectiveness of OPCAB, mostly as a bridge to safe non-cardiac surgery that can be crucial for long-term survival. There are no guidelines, about the correct treatment of this double pathology. Searching the international bibliography, a patient with colorectal cancer and cardiac tumor underwent anterior resection for sigmoid colon cancer and the cardiac operation was performed two weeks after the colorectal intervention with good results [14]. In a report of 14 patients witch needed colectomy and cardiac operation, simultaneous intervention was performed in 3 patients, 11 patients were operated consecutively with a similar outcome [15]. Valve replacement with the use of extracorporeal circulation and colectomy has also been described. In patients with surgical indications for both cardiac surgery and a malignant neoplasm, cardiac surgery should generally be performed first; mitral valve replacement and a Hartmann's operation were performed simultaneously [16]. Cardiovascular pathologies and cancer often coexist. In a Japanese case report paper, simultaneous operations were scheduled because of the hazardous collaterals of the coronary arteries, rapid expansion of an abdominal aortic aneurysm, and subileus due to the presence of cancer in a 68-year-old man. The patient underwent simultaneous off-pump coronary artery bypass grafting, abdominal aortic aneurysm repair with a tube graft Miles' operation with total mesorectal excision. 12 months after the operation, neither myocardial ischemic symptoms nor recurrence of cancer came to the fore [17]. Mistiaen et al in a study of 8620 patients referred for cardiac surgery, 205 of them had a history of documented malignant tumour. Fatal incidents of t tumour were seen if the period between the occurrence of the malignant tumour and cardiac surgery was short. Other unfavourable factors were: decreased left ventricular function, chronic obstructive pulmonary disease and high age [18]. Off-pump coronary revascularization and nephrectomy due to malignancy has recently been reported in literature. The patient outcome was uneventful and the follow-up 17 months, did not reveal any sings of recurrence neither symptoms of angina [2]. It has been described in the bibliography a series of combined open heart surgery and lung intervention (lobectomy or wedge lung resection) simultaneously. According to reports, heart and lung operation can be performed without increased mortality and/or morbidity. Treatment at the same time prevents the need of a second intervention with both a financial benefit and an excellent outcome [19,20]. In some cases coexistence of cancer and cardiac disease is unknown. Bonde et al described a 68-year-old male who presented chronic anaemia; an initial investigation revealed colonic polyps, but anaemia persisted after polypectomy. Further investigation revealed a right atrial myxoma arising from the Eustachian valve and prolapsing into the right ventricle through the tricuspid valve. After successful resection of the lesion, hematological,parameters returned to normal [21]. As we can see, little is known about the order of treatment in case of need cardiac intervention and concomitant colorectal cancer operation. A case report study describes a patient who underwent emergent resection of a cardiac mass under cardiopulmonary bypass which followed a successful resection of a colorectal mass two weeks later [13]. In elderly patients affected from coronary artery disease (CAD) and cancer, angioplasty and percutaneous transluminal coronary angioplasty (PTCA) may be an essential way of treatment prior to colectomy. In these patients of advanced age and hemorrhagic cancer, conventional coronary aorta bypass grafting (CABG) using extracorporeal circulation, as well as coronary stenting requiring antiplatelet therapy, is regarded avoidable. In addition, when OPCAB technique is feasible, other serious complications of CPB such as neurocognitive disorders and pathophysiological changes that contribute to multi-organic failure are avoided, the morbidity and mortality discrease [22,23]. Clinically, patients seem to benefit from the avoidance of the extracorporeal bypass machine and all the major pathophysiological changes that seem to be related with it, including coagulopathies, functional disturbances of heart itself, as well as renal and pulmonary dysfunction [2,4,22-24]. The hypothesis that a systemic inflammatory reaction takes place after the use of CPB, could explain most of the adverse effects influences the lung. On the other hand, the release of various proinflammatory cytokines such as TNF-α, IL-1, IL-2, IL-6, IL-8 during CPB can increase the entrapment of neutrophils in the pulmonary capillaries [24]. Taking into consideration these pathophysiological mechanisms of injury, the OPCAB technique firstly because of the severe coronary artery disease and secondly the colectomy due to hemorrhagic left colon cancer, was performed simultaneously. As a supreme tool the off pump technique of coronary artery bypass grafting (OPCAB), facilitated the one stage cardiac revascularization and colectomy to be done safely at the same time. However, the beating heart coronary revascularization without extracorporeal circulation (OPCAB) and colectomy simultaneously, is proposed [25]. The unknown but presumably reduced life expectancy of patients with malignant neoplasms may dissuade surgeons from performing necessary coronary and valvular heart operations. There is also concern for recrudescence of cancer as a result of an impaired immune system after cardiopulmonary bypass [26]. According to literature, open heart operations may be performed in patients with cardiac symptoms only in the absence of tumour recurrence. Canver and colleagues presented their experience suggesting that cardiac operations in selected patients with previously treated cancer are safe and offer clinical improvement at a reasonable operative risk [26]. In the international bibliography are however described cardiac metastasis from colorectal cancer [14,27]. Some authors reported cases of cardiac metastasis of a rectal adenocarcinoma that infiltrated the right ventricle; partially obstructed its outflow tract [27], or the right atrium [14]. Simultaneous active intestinal bleeding and acute heart ischemia create a troublesome environment about patient's surgical approach. In chronic complicated cases, composed of more than one pathologic entities, it is of great acceptance to deal firstly with diseases that affect the cardiopulmonary system and then to provide cure to other chronic situations such as malignancy. In our case OPCAB revascularization was totally indicated. Patient's physical status was good. Spirometry values were positive indicators that the patient will overcome the side effects of median sternotomy. No accompanied chronic diseases were involved in his postoperative care. Thus we consider that the subject was a good candidate for a simultaneous surgical treatment of both pathologies. Although the patient underwent a very serious operation he was discharged, 10 days after surgery with no postoperative complications. A digital CT coronary angiography 8 months later, verified that the two bypass grafts were functional (figure 4). There were no symptoms of angina or signs of cancer recurrence.

## Conclusion

Off-pump coronary artery bypass surgery offers a proper solution in cases of coexisting oncologic and heart diseases. The option of operating two diseases synchronously provides a postoperative course free of complications bound to cardiopulmonary bypass. OPCAB secures patient's survival and does not disturb the oncologic profile of the coexisting malignancy.

## Competing interests

The authors declare that they have no competing interests.

## Authors' contributions

All authors have made substantial contributions to the conception and design, or acquisition of data, or analysis and interpretation of data; have been involved in drafting the manuscript or revising it critically for important intellectual content, and have given final approval of the version to be published.
